# Sequential Use of CO_2_ Laser Prior to Nd:YAG and Dye Laser in the Management of Non-Facial Warts: A Retrospective Study

**DOI:** 10.3390/medicina58010115

**Published:** 2022-01-12

**Authors:** Luigi Bennardo, Gaia Fasano, Federica Tamburi, Elena Zappia, Francesco Rizzuto, Steven Paul Nisticò, Giovanni Cannarozzo

**Affiliations:** 1 Department of Health Sciences, Magna Graecia University, 88100 Catanzaro, Italy; luigibennardo10@gmail.com (L.B.); fasano.gaia@gmail.com (G.F.); federica.tamburi@gmail.com (F.T.); elena.zappia@hotmail.it (E.Z.); francescorizzuto22@libero.it (F.R.); 2 Department of Systems Medicine, Tor Vergata University, 00173 Rome, Italy; drcannarozzo@gmail.com

**Keywords:** CO_2_ laser, Nd:YAG laser, dye laser, warts

## Abstract

*Background and Objectives*: Warts are benign lesions of viral etiology characterized by a hyperkeratotic appearance tending to spread across the skin surface. Various treatments have been proposed to manage this condition, such as acids, imiquimod, photodynamic therapy, cryotherapy, and various lasers. *Materials and Methods:* In this paper, we describe a combination protocol using CO_2_ laser prior to Nd:YAG laser for lesions interesting the palmoplantar areas or dye laser for lesions on other skin surfaces in the management of non-facial warts resistant to traditional therapies. In total, 34 patients with 103 warts suffering from wart infection resistant to traditional therapies treated from 1 January 2019 to 1 June 2020 were retrospectively enrolled at the Dermatological Unit of Magna Graecia University (Catanzaro, Italy). Two dermatologists measured clinical results, classifying lesions with complete resolution, partial resolution, or non-responding. Patients at four months follow-up were asked to evaluate their degree of satisfaction with a visual analog scale (VAS). *Results:* Almost all patients reported the complete resolution of lesions, with no patient reporting scarring. Five patients reported hypopigmentation in the treated areas. The mean satisfaction level was high. Only three patients experienced a relapse of the condition. *Conclusions:* Using a vascular laser following a CO_2_ superficial ablation of warts may help reduce the risk of scarring and decrease the incidence of relapses for lesions resistant to traditional therapies. Therefore, more extensive studies will be necessary to confirm the obtained results.

## 1. Introduction

Warts (verrucae vulgaris) are benign lesions caused by several types of human papillomavirus (HPV), a DNA virus from the Papillomaviridae family, which affects about 10% of the population with a high prevalence in school-aged children. The infection may be localized in the squamous epithelium of the mucosa or the skin [1]. Transmission can be direct or indirect, especially in the interruption of the epithelial barrier, as in minor trauma. Common warts appear as an indurated papule with an irregular hyperkeratotic surface and vegetations; they can be single or multiple, small or large (several centimeters) [2].

Histological examination shows acanthosis, hyperplasia, papillomatosis, orthokeratosis, hypergranulosis, and thrombosed capillaries in dermal papillae; the presence of koilocytic cells is pathognomonic [3]. Different treatments can be used for the therapy of common warts; they must be adapted according to the number of warts, their size, localization, symptoms, the patient’s preference, cost, the status of the immune system, and previous treatment modalities. The cosmetic discomfort and the risk of translocation in other areas are the most frequent indication of treatment [4]. Therapies are not always effective, especially in immunocompromised patients, but it is necessary to start with the least painful and expensive treatment that leaves no scars reserving the most expensive and painful treatment in case of recurrent warts. Sometimes, warts resolve spontaneously in several months or years. First-line therapy is based on chemical topic agents such as salicylic acid, retinoic acid, podophyllin, topical 5-fluorouracil, and imiquimod; they require several weeks or months and can often be unsuccessful [5]. Cryotherapy, diathermocoagulation, and surgical curettage are more aggressive and can cause moderate/severe scars, tissue damage, and adverse effects [6]. The combination of lasers and conventional therapies such as immunomodulators, keratolytic agents, or photodynamic therapy is advantageous in cases of recalcitrant warts and immunosuppressed patients. Different types of laser therapy can be used, such as carbon dioxide (CO_2_) laser, neodymium-doped yttrium aluminum garnet (Nd:YAG) laser, pulsed dye laser (PDL), or a combination of them. Usually, these devices have been proposed alternatively and before surgery when all other local treatments failed [7,8]. In this paper, we report, for the first time, a combined therapy using a CO_2_ laser prior to Nd:YAG or dye laser in the management of recalcitrant warts.

## 2. Materials and Methods

Patients treated for recalcitrant non-facial warts not responding to traditional therapy with this new combined technique between 1 January 2019 and 1 June 2020 at the Unit of Dermatology of Magna Graecia University were retrospectively enrolled. Patients reporting hypersensitivity to light or reporting the use of sulfonamides, sulfonylureas, phenothiazines, and contraceptives, being pregnant, breastfeeding, or malignant tumors were excluded from the study. All patients signed informed consent on the risk of the procedure. The treatment consisted of the first session of CO_2_ laser (Smartxide Punto, DEKA M.E.L.A., Calenzano, Italy) in super-pulsed mode, with a focused beam of 0.1–0.2 mm diameter, power 0.3–0.8 W, frequency 10 Hz, with the intent to remove the superficial layer of the epidermis, exposing the superficial dermis but without causing bleeding. Then, a second session with long-pulsed Nd:YAG laser (Luxea, DEKA M.E.L.A., Calenzano, Italy) for lesions interesting the palmoplantar region (90–120 J/cm^2^, 5 mm spot size, slightly defocused, double pulse 5 ms–15 ms with a 10 ms interval performing multiple passes), and with a 595 nm dye laser (Synchro Vasq, DEKA M.E.L.A., Calenzano, Italy) for all other regions (10 mm size spot, fluence 9–10 J/cm^2^, and frequency 0.5/s, performing multiple passes) was performed on the treated spot, in order to reduce the vascularization to the treated area. The end-point of the treatment was the darkening of the lesion. Subcutaneous injection of lidocaine was proposed when patients suffered pain during the procedure. After the treatment session, topical fusidic acid was applied to the lesion twice a day for one week. From the second week, a 20% salicylic acid cream in the evening and 40% urea in the morning were applied for the other three weeks up to clinical follow-up. A clinical follow-up was performed one month after therapy to evaluate lesions’ disappearance. Four months after initial therapy, a second follow-up was performed to evaluate possible relapses. During both clinical follow-ups and before treatments, pictures of the lesions with the same ambient light and shooting parameters were taken. A visual analog scale (VAS) from 1 to 10 to measure patient satisfaction was administered to subjects during the last clinical follow-up. Statistica14.0 (TIBCO Software, Palo Alto, CA, USA) software was used for data analysis (mean, standard deviations, and rate calculations).

## 3. Results

**Figure 1:**
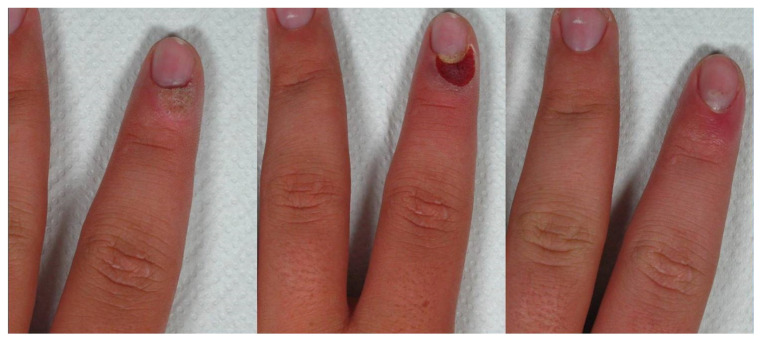
Patient n. 3 before treatment (**left**), during laser therapy (**central**), and 4 months after treatment (**right**).

**Figure 2:**
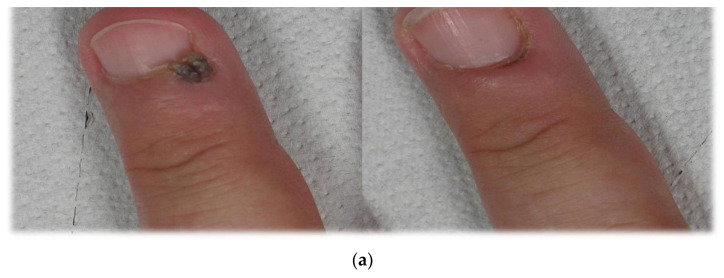

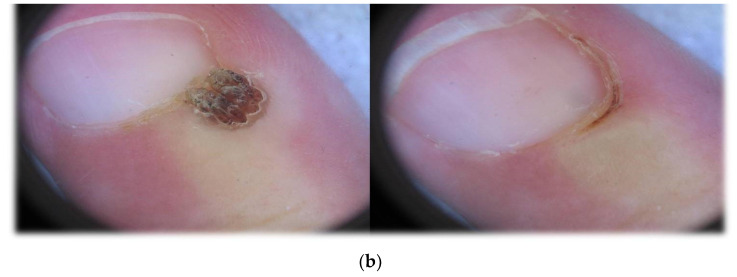
(**b**) Patient n. 20 before treatment (**left**) and 1 month after treatment (**right**) dermatoscopic view; (**a**) patient n. 20 before treatment (**left**) and 1 month after treatment (**right**).

**Figure 3:**
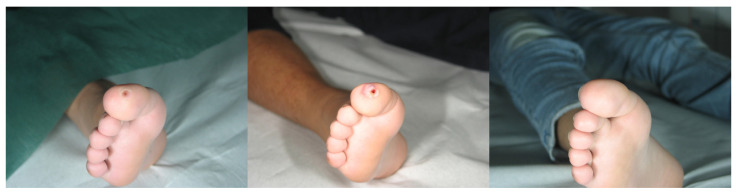
Patient n. 6 before treatment (**left**), immediately after combined treatment (**central**), and 4 months after treatment (**right**).

**Figure 4:**
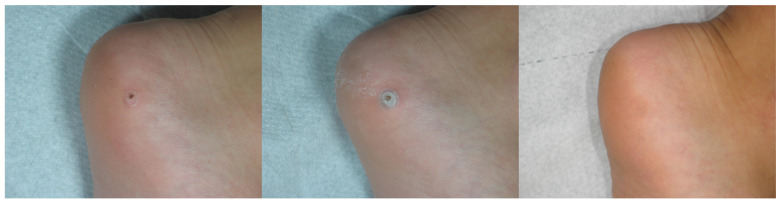
Patient n. 27 before treatment (**left**), immediately after combined treatment (**central**), and 4 months after treatment (**right**).

**Figure 5:**
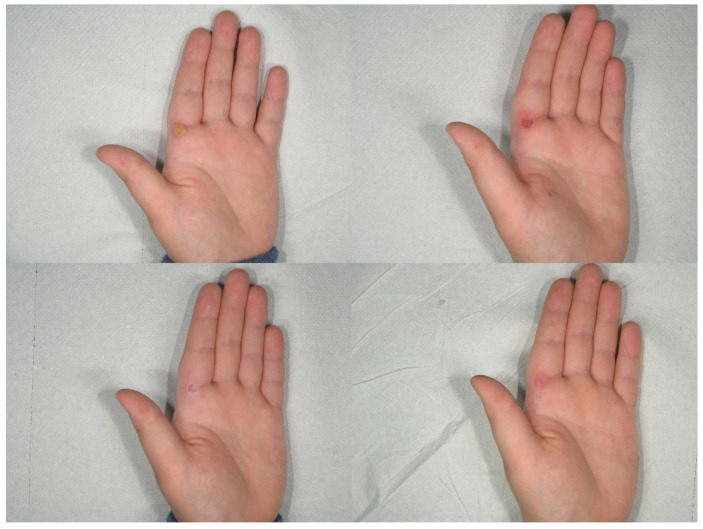
Patient n. 19 before treatment (**upper left**), immediately after CO_2_ treatment (**upper right**), immediately after combined treatment (**lower left**), and 1 month after treatment (**lower right**).

In total, 34 patients, comprising 16 males and 18 females (mean age 44.24 ± 21.24), with a total number of 103 warts were enrolled in the study. Of those, 16 patients had warts on the palmoplantar region, 18 patients on other body areas. In addition, 26 patients suffered pain during the procedure, and local anesthesia was necessary, especially for the palmoplantar area. At one month follow-up, 32 patients reported complete resolution of the condition, with 2 patients from the palmoplantar group reporting partial resolution. No severe side effects were reported. At four months of follow-up, three patients experienced a relapse of the condition that was subsequently treated with the same protocol, reaching complete disappearance. Five patients reported hypopigmentation of the treated area. The reported VAS score for patients was very high (9.29 ± 0.97) (Figure 1, Figure 2, Figure 3, Figure 4 and Figure 5). Patient characteristics are reported in Table 1. A treatment protocol diagram is reported in Table 2.

## 4. Discussion

The CO_2_ laser is an ablative laser that uses water as a chromophore target [9,10]. It was the first laser used for warts treatment [11].

The CO_2_ laser can be used in focused mode or non-focused mode with stimulation of immunity of infected keratinocytes [12].

It is necessary to gradually vaporize layer by layer and remove the burned tissues with a cotton-containing physiological solution during the procedure. The treatment can be repeated at intervals of 30–45 days [13].

The advantages of using the CO_2_ laser are the precision in the depth of ablation that does not damage the adjacent tissues, the quickness in case of multiple lesions, and the absence of bleeding [14].

Adverse effects are pain, edema, infections, hyperpigmentation, scars, and dystrophy in the treatment of periungual warts [7].

Takac investigated the efficacy of surgical laser, compared with other conventional treatments; a CO_2_ surgical laser has been used with high efficacy to vaporize and resect the root of warts. No significant side effects were reported; healing occurred painlessly and without scarring, suggesting this method has numerous advantages over conventional therapies [15]. Different authors reported the use of CO_2_ laser in immunocompetent patients with good results [16,17].

The CO_2_ laser can also be used to treat challenging recalcitrant warts in immunosuppressed patients. In immunocompromised patients, HPV is a risk factor implicated in the onset of non-melanoma skin cancer. Specifically, squamous cell carcinoma has been linked with this infection [18,19]; these patients often present with multiple warts that are unresponsive to conventional treatments and often persist and spread. The presence of 10 or more warts is a high-risk factor for the onset of squamous cell carcinoma (SCC) [20]. The duration of immunosuppression also increases the incidence of these tumors; on average, 25% of patients with a previous kidney transplant develop SCC after about 10 years from the start of immunosuppressive therapy [21].

Läuchli et al. treated 13 immunocompromised patients with multiple relapsing warts; they used a CO_2_ laser with fluences of 3–10 W in super pulsed mode. Response rates were in line with those of non-immunocompromised patients undergoing the same CO_2_ laser treatment [21]. Additionally, fractional laser, generating columns of heat in the epidermis [22], has been proposed to enhance drug penetration, as already experimented with for other conditions [23].

We propose that CO_2_ laser may be used to substitute keratolytic therapies to expose superficial dermis, and then vascular lasers, according to the interested area, may be used to isolate the area, reduce the blood flow, and lower the risk of relapses. The use of keratolytic creams in the weeks following the procedures may help eliminate the necrotic debris following the action of vascular lasers on the exposed lesions. For this reason, it is fundamental to expose the superficial dermis with the CO_2_ laser without causing bleeding during this procedure, in order to hit the small vessels with vascular lasers safely. Given the longer wavelength of Nd:YAG laser, we used it on thicker areas, such as palmoplantar regions, while PDL, with a shorter wavelength and a minor ability to penetrate through the skin, was used in all the other areas.

PDL produces pulses of visible light at a 585 or 595 nm wavelength. The PDL using the wavelength of 585 nm can hit the capillaries, so the vaporization and coagulation should block the replication of warts and promote their healing, but hyperpigmentation is a possible adverse side effect [24].

The mechanism of action of the PDL is the selective photothermolysis of dermal blood vessels without damaging the surrounding structure, reducing the risk of scar formation [25].

Photothermolysis of dermal blood vessels stimulates the release of inflammatory cytokines and enhances a cellular immune system response, which contributes to the elimination of the virus; the immunomodulating properties also support the wart’s healing [26,27].

To avoid the onset of side effects such as scars and pigment changes, it is necessary to use the PDL with low fluences and as few impulses as possible. On the contrary, the palmar and plantar areas have a high healing capacity and can be treated with higher fluences and more impulses. The cycle is repeated every 15–20 days until complete resolution. [28].

The advantages of using the PDL are rapid healing and the absence of pain, and nail dystrophy in periungual warts. PDL can be used to treat recalcitrant warts in children [29].

Currently, the most used treatment by pediatricians for warts in children is cryotherapy, which has a response rate of 40–82%. In case of recurrences, different therapies are available: topical or systemic immunotherapy, intralesional bleomycin, surgical excision, curettage, and cautery or lasers, especially carbon dioxide (CO_2_) and pulsed dye laser (PDL) [30,31].

Sethuraman et al. described 61 children treated with PDL. A 5–7 mm spot was used at 7 J/cm^2^, and an average of three pulses per wart. Follow-up lasted 1–5 years, and the overall clearance rate was 75%, with only 25% partial responses; most patients required about three treatments, while 13% needed only one treatment. Side effects were minimal (hypopigmentation, hyperpigmentation, itching, scars), compared with a CO_2_ laser (postoperative pain, bleeding, scarring, and hypopigmentation) [32,33]. Therefore, PDL is a safer, more effective, and well-tolerated treatment for children than cryotherapy and CO_2_ laser [34].

In contrast, as described in other studies, Huilgol et al. reported that PDL treatment in their patients resulted in partial remission and symptom reduction without complete resolution. Different factors could explain this result (immune status, warts’ number, location, and persistence, number of laser sessions performed) [35].

Nd:YAG is a crystal used as a laser medium for solid-state lasers; it has a wavelength of 1064 nm and a deeper penetration level of skin tissue than other types of lasers [36,37].

In Q-switched mode, Nd:YAG produces two wavelengths—one in the infrared range (1064 nm) and the second beam of 532 nm wavelength, which is helpful for superficial skin lesions [38,39].

Hemoglobin has two absorption peaks: one greater at the wavelength of 585–595 nm and another smaller in the wavelengths between 800 and 1000 nm [40].

By modulating the pulse duration and energy density, it is possible to obtain two different effects on the target tissue: coagulation (photothermal effect) and destruction (photomechanical effect). Monochromatic light is absorbed by hemoglobin (targeted tissue chromophores) and converted to thermal energy; this determines the destruction of the tissue [41,42]. The 1064 nm Nd:YAG laser can target the red structures such as hemoglobin in blood vessels, targeting the capillaries present inside warts [43].

In the days following the treatment, it is possible to observe the changes through a microscopic evaluation: Separation of the dermal–epidermal junction, epidermal necrosis, RBC extravasation, and destroyed blood vessels with dense inflammatory infiltrate in the dermis will be obtained [44].

The mechanism of Nd:YAG laser’s function is probably due to an interruption of the supply of vital substances for the wart, or it is linked to the destruction of the epidermal cells that contain HPV. An advantage of using Nd:YAG laser vs. conventional therapies such as cryotherapy is the greater ability to eliminate HPV DNA after treatment [45].

Unlike the PDL, its target does not include the melanin of the dermal–epidermal junction, so there is no hyperpigmentation as a side effect but can cause blood vessel rupture with purpura 5–7 days after the treatment [46].

In Han’s study, 348 patients with untreated or relapsed warts were treated with Nd:YAG laser. The parameters used were spot size 5 mm, pulse duration 20 ms, fluence 200 J/cm^2^. Biopsy after treatment showed destruction of blood vessels. After the first treatment, 64% of warts were wholly cleared, while 96% were cleared after four treatment sessions. The average number of treatments required for clearance was 1.49. Verruca Vulgaris had a better response than deep palmoplantar warts, while only 3.27% of warts relapsed over the next 2–10 months. The most frequent side effects were pain (82%), transient numbness (15%), hemorrhagic bullae (7%), hyperpigmentation (5%), and hypopigmentation (4%). Han et al. concluded that the long-pulsed Nd:YAG laser is a safe and effective treatment for removing or reducing warts and does not depend on patient compliance [46]. El-Mohamady et al. compared the use of the PDL and Nd:YAG laser to treat plantar warts. In their study, 46 patients with multiple and relapsing warts were treated after topical or local anesthesia and a surgical curettage with a scalped blade [47].

In each patient, half of the warts were treated with PDL, using a spot size of 7 mm, 8 J, and a pulse duration of 0.5 ms, while the other half were treated with Nd:YAG laser using a spot size of 7 mm, 100 J, and a pulse duration of 20 ms. With PDL, 73.9% of patients achieved a complete remission rate (34 patients), while with Nd:YAG laser, 78.3% (36 patients) [47].

The incidence of side effects was higher in patients treated with Nd:YAG laser (43.5%) than in patients treated with PDL (8.7%); specifically, hematoma (28.3% vs. 2.2%), bacterial infections (10.9% vs. 4.3%), and erythema (4.3% vs. 2.2%) incidence rates were higher (20). On the contrary, the number of relapses was higher in patients treated with PDL (13% vs. 8.7%) (20). Therefore, PDL is safe and associated with fewer side effects but requires a greater number of treatments for the complete resolution of the lesions; on the contrary, Nd:YAG laser is more effective and requires fewer treatments but is associated with more pain and a greater incidence of side effects [47].

Zorman et al. used the Nd:YAG laser without anesthesia to treat warts on 85 patients. The treatment is safe and effective using alternative techniques such as ice cubes every 3–4 laser pulses or cold air to reduce pain and thermal damage of the surrounding tissues. None of the patients experienced significant side effects following the procedure; 4 patients had blisters, and 14 patients reported slight pain in the following days; no patient reported hypo/hyperpigmentation or scarring. Patients reported that the perceived pain was similar to cryotherapy’s procedures, with minor discomfort in the following days. In their experiences, topical anesthesia did not reduce the pain significantly more than placebo, with response rates similar or higher than those obtained with conventional therapies [48].

Khattab et al. described the combined treatment with long-pulsed Nd:YAG laser and potassium hydroxide (KOH) to treat recalcitrant warts. In total, 38 patients with 132 lesions were recruited, from which 66 lesions were treated with a long-pulsed Nd:YAG laser every four weeks, while the other 66 also added a 10% KOH application once daily at night. In both groups, after the first treatment, there was a reduction in wart size, with no statistically significant difference; after the second treatment, LP Nd:YAG laser plus KOH showed higher effectiveness. The mechanism of action is due to the ability of KOH to reduce hyperkeratosis of warts by favoring laser penetration [49].

Only a few cases of combined laser therapies for the treatment of warts have been reported in the literature. The first description of a successful technique with the combination of PDL and CO_2_ laser in treating recalcitrant warts was proposed by Mixer et al. [50]. Based on Geronemus et al.’s data trial, the use of the PDL alone has fewer side effects than the combination with the CO_2_ laser as described by Mixter et al. [50,51]. The combined use of the two lasers requires a prolonged healing time with the possibility of pain and scarring in the following days. The main side effect of using PDL is the appearance of a purpuric area that resolves within 7–10 days.

According to Geronemus et al., the treatment with PDL is effective only if fluences of about 7 J/cm^2^ are used [51]. To date, no further studies described the combined use of multiple lasers for wart’s treatment. The efficacy of combined laser therapies has been described to treat various pathologies.

The combined use of the Nd:YAG laser in association with PDL has been described for the treatment of melasma [52], tattoo removal [53], hypertrophic scar [54], capillary malformation [55], infantile hemangiomas [56], port-wine stains [57], PHACES syndrome [58], Mibelli’s angiokeratoma [59] and cutaneous symptoms of connective tissue disease [60]. The combined use of the CO_2_ laser in association with PDL has been described for the treatment of flap necrosis after surgery [61], keloids [62], and giant epidermal nevus [63].

The ablative CO_2_ laser requires fewer treatments to achieve complete healing than non-ablative lasers such as PDL and Nd:YAG laser. Among non-ablative lasers, Nd:YAG laser requires fewer sessions than PDL. The Nd:YAG laser is more effective in treating plantar warts [64]. PDL treatment has fewer side effects than Nd:YAG laser treatment, but it is less effective. The combination of topical or keratolytic therapies may be helpful before laser treatment to achieve a better response [4].

## 5. Conclusions

This article proposed a combined treatment using a first pass of CO_2_ laser to expose the viral lesions, followed by a second pass of vascular laser to reduce the vascular afflux to the area, necrotize viral warts, and reduce the relapse rate. Pulsed dye laser was used in the area where the skin was thin, and Nd:YAG in the areas with thicker skin, with an optimal result; the response rate was very high, and the relapse rate was very low, suggesting this combined treatment as a valid alternative in recalcitrant and treatment-resistant warts. It was fundamental to associate keratolytic creams in the weeks following the procedure to eliminate any necrotic debris following vascular laser treatment. Limitations of this study included the relatively low number of participants and the absence of a control group using only salicylic acid and urea to manage warts.

Vascular lasers are a very operator-dependent procedure, so it is fundamental for the physician to be adequately trained to improve the results and reduce side effects. In addition, further studies and trials are necessary to confirm the findings of this study, and a prospective trial comparing this new combination with single techniques will be required to validate the results obtained by this paper.

## Figures and Tables

**Table 1:** Patient characteristics.

Patient Number	Sex	Age	Number of Warts	Location	Resolution	Relapse	Side Effects	VAS Score	Anesthesia
1	M	71	1	PP	T			10	Yes
2	M	9	4	O	T			10	No
3	F	29	3	O	T			10	Yes
4	F	38	5	PP	T		H	8	Yes
5	M	68	4	O	T			10	Yes
6	F	12	1	PP	T			10	Yes
7	M	28	4	O	T			9	No
8	F	67	3	O	T		H	7	Yes
9	M	23	2	O	T			9	Yes
10	F	65	1	PP	P			7	Yes
11	F	14	4	O	T	R		8	No
12	M	45	3	PP	T			10	Yes
13	F	34	7	O	T			10	Yes
14	F	22	3	PP	T			10	Yes
15	F	74	3	O	T			10	Yes
16	M	67	1	PP	T		H	9	Yes
17	F	68	1	PP	P			10	Yes
18	M	25	5	O	T			10	No
19	M	54	1	PP	T			10	Yes
20	F	52	2	O	T			10	No
21	M	78	1	PP	T	R		7	Yes
22	F	15	6	O	T			9	Yes
23	M	19	3	PP	T	R		9	Yes
24	F	64	5	O	T			10	No
25	F	43	1	PP	T		H	10	Yes
26	M	37	4	O	T			8	Yes
27	F	34	1	PP	T			9	Yes
28	M	25	3	O	T		H	10	Yes
29	F	27	4	O	T			9	No
30	F	58	1	PP	T			9	Yes
31	M	67	4	O	T			10	Yes
32	M	65	3	O	T			9	No
33	M	64	4	PP	T			10	Yes
34	F	43	5	PP	T			10	Yes

M: male, F: female, PP: palmoplantar region, O: other regions, T: total, P: partial, R: relapse, H: hypopigmentation.

**Table 2:** Treatment protocol.

First session of CO_2_ laser in super-pulsed mode, with a focused beam of 0.1–0.2 mm diameter, power 0.3–0.8 W, fre-quency 10 Hz, with the intent to remove the superficial layer of the epidermis, exposing the superficial dermis but without causing bleeding.
Right after CO_2_ laser, a second session with long-pulsed Nd:YAG laser for lesions interesting the palmoplantar region (90–120 J/cm^2^, 5 mm spot size, slightly defocused, double pulse 5–15 ms with a 10 ms interval performing multiple passes), and with a 595 nm Dye laser for all other regions (10 mm size spot, fluence 9–10 J/cm^2^, and frequency 0.5/s, per-forming multiple passes) was performed on the treated spot, in order to reduce the vascularization to the treated area.
After the combined treatment, topical fusidic acid was applied to the lesion twice a day for one week.
From the second week, a 20% salicylic acid cream in the evening and 40% urea in the morning were applied for the other three weeks up to clinical follow-up.
A first clinical follow-up at one month evaluated lesion disappearance.
A second clinical follow up at four months evaluated relapses.

## Data Availability

Data available upon request from the corresponding author due to privacy issues.
